# Prediction of hematoma expansion in spontaneous intracerebral hemorrhage using a multimodal neural network

**DOI:** 10.1038/s41598-024-67365-3

**Published:** 2024-07-16

**Authors:** Satoru Tanioka, Orhun Utku Aydin, Adam Hilbert, Fujimaro Ishida, Kazuhiko Tsuda, Tomohiro Araki, Yoshinari Nakatsuka, Tetsushi Yago, Tomoyuki Kishimoto, Munenari Ikezawa, Hidenori Suzuki, Dietmar Frey

**Affiliations:** 1 Charité Lab for Artificial Intelligence in Medicine, Charité Universitätsmedizin Berlin, Charitéplatz 1, 101117 Berlin, Germany; 2 Department of Neurosurgery, Mie University Graduate School of Medicine, 2-174 Edobashi, Tsu, Mie 5148507 Japan; 3 Department of Neurosurgery, Mie Chuo Medical Center, 2158-5 Myojin-Cho, Hisai, Tsu, Mie 5141101 Japan; 4 Department of Neurosurgery, Matsusaka Chuo General Hospital, 102 Kobo, Matsusaka, Mie 5158566 Japan; 5 Department of Neurosurgery, Suzuka Kaisei Hospital, 112-1 Ko-Cho, Suzuka, Mie 5138505 Japan; 6 Department of Neurosurgery, Charité Universitätsmedizin Berlin, Charitéplatz 1, 10117 Berlin, Germany

**Keywords:** Intracerebral hemorrhage, Hematoma expansion, Prediction, Multimodal neural network, Cerebrovascular disorders, Stroke

## Abstract

Hematoma expansion occasionally occurs in patients with intracerebral hemorrhage (ICH), associating with poor outcome. Multimodal neural networks incorporating convolutional neural network (CNN) analysis of images and neural network analysis of tabular data are known to show promising results in prediction and classification tasks. We aimed to develop a reliable multimodal neural network model that comprehensively analyzes CT images and clinical variables to predict hematoma expansion. We retrospectively enrolled ICH patients at four hospitals between 2017 and 2021, assigning patients from three hospitals to the training and validation dataset and patients from one hospital to the test dataset. Admission CT images and clinical variables were collected. CT findings were evaluated by experts. Three types of models were developed and trained: (1) a CNN model analyzing CT images, (2) a multimodal CNN model analyzing CT images and clinical variables, and (3) a non-CNN model analyzing CT findings and clinical variables with machine learning. The models were evaluated on the test dataset, focusing first on sensitivity and second on area under the receiver operating curve (AUC). Two hundred seventy-three patients (median age, 71 years [59–79]; 159 men) in the training and validation dataset and 106 patients (median age, 70 years [62–82]; 63 men) in the test dataset were included. Sensitivity and AUC of a CNN model were 1.000 (95% confidence interval [CI] 0.768–1.000) and 0.755 (95% CI 0.704–0.807); those of a multimodal CNN model were 1.000 (95% CI 0.768–1.000) and 0.799 (95% CI 0.749–0.849); and those of a non-CNN model were 0.857 (95% CI 0.572–0.982) and 0.733 (95% CI 0.625–0.840). We developed a multimodal neural network model incorporating CNN analysis of CT images and neural network analysis of clinical variables to predict hematoma expansion in ICH. The model was externally validated and showed the best performance of all the models.

## Introduction

Spontaneous intracerebral hemorrhage (ICH) is a severe form of stroke with a high mortality rate^1^. In its early clinical stage, 20–30% of patients experience hematoma expansion, leading to neurological deterioration or the need for surgical treatment^2,3^. Accurate stratification of expansion risk on admission therefore guides patient management, including admission to the intensive care unit and transfer from remote areas to specialist care.

CT image analysis plays an important role in predicting hematoma expansion. Several CT findings, such as blend sign or intrahematoma hypodensities, have been identified as potential indicators of hematoma expansion^4–10^. Scoring systems incorporating both CT findings and clinical variables have also been proposed^11–13^. However, their sensitivity and C-statistics have been found to be low to moderate in external validation and remain unsatisfactory for clinical application^14–16^. Machine learning models based on CT findings and clinical variables have been proposed, demonstrating superior predictive ability compared to the traditional scoring systems^14,17^. However, a major drawback of both the scoring systems and machine learning models is that prediction requires CT findings to be evaluated by human experts.

Recently, a convolutional neural network (CNN) has been applied to image analysis in ICH^18–23^, with superior predictive ability of hematoma expansion compared to previously proposed CT findings and scoring systems^18^. CNNs can directly interpret the image data without the evaluation of human experts, overcoming the drawbacks of the scoring systems and machine learning models. Furthermore, in other fields, a multimodal neural network that combines CNN analysis of images and neural network analysis of clinical information such as age, sex, medical history, etc. has been introduced for disease type or risk classification tasks, which has demonstrated better performance than CNN analysis of images alone^24,25^.

Here, we hypothesized that it would be feasible to create a multimodal neural network model incorporating CNN analysis of CT images on admission and neural network analysis of clinical variables to predict hematoma expansion in acute ICH, and that the model would outperform CNN analysis of CT images alone and be superior to machine learning analysis of CT findings and clinical variables.

## Methods

### Patients

**Figure 1:**
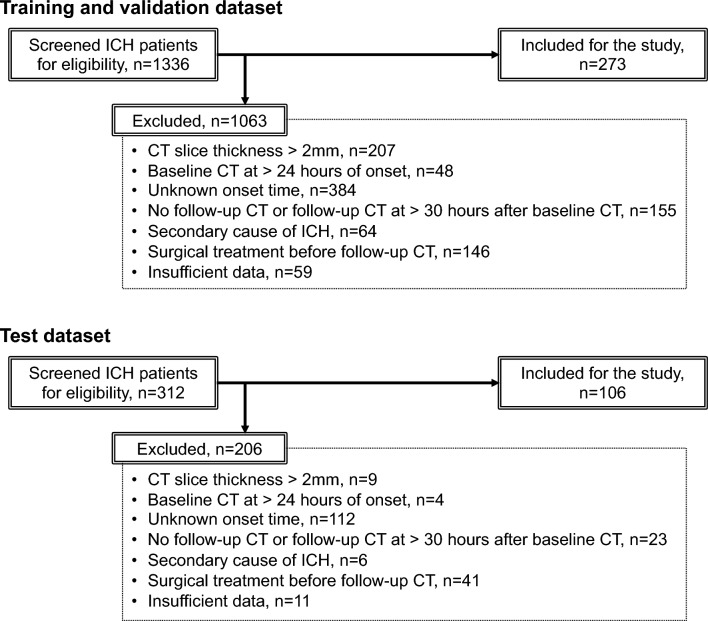
Flow diagram of patient selection. ICH = intracerebral hemorrhage.

We retrospectively reviewed consecutive patients with spontaneous ICH, aged ≥ 18 years, admitted to a university hospital and three community hospitals in Japan between January 2017 and December 2021. Patients were included if they underwent a baseline CT scan with a thickness of 2.0 mm or less within 24 h of onset and a follow-up CT scan within 30 h of the baseline CT scan. Patients were excluded if they had a secondary cause of ICH (e.g., tumor, aneurysm, arteriovenous malformation, arteriovenous fistula, and hemorrhagic transformation of ischemic infarction), head trauma, or surgical hematoma evacuation before the follow-up CT scan, or if they had missing data. Patients from a university hospital and two community hospitals were assigned to training and validation dataset, while patients from the other community hospital were assigned to a test dataset (Fig. 1).

This study was approved by the following institutional review boards: Mie Chuo Medical Center institutional review board [permit number: MCERB-202321], Matsusaka Chuo General Hospital institutional review board [permit number: 325], Suzuka Kaisei Hospital institutional review board [permit number: 2020-05], and Mie University Hospital institutional review board [permit number: T2023-7]. Because this was a retrospective study, separate informed patient consent was waived by the following institutional review boards: Mie Chuo Medical Center institutional review board [permit number: MCERB-202321], Matsusaka Chuo General Hospital institutional review board [permit number: 325], Suzuka Kaisei Hospital institutional review board [permit number: 2020-05], and Mie University Hospital institutional review board [permit number: T2023-7]. All study protocols and procedures were conducted in accordance with the Declaration of Helsinki. This manuscript was prepared according to the standards for reporting of diagnostic accuracy (STARD) statement.

### Clinical variables

Demographic and clinical data on admission were collected. The following variables were recorded: age, sex, medical history (hypertension, diabetes mellitus, dyslipidemia, ICH, cerebral infarction, and ischemic heart disease), anticoagulant use, antiplatelet use, systolic and diastolic blood pressure, Glasgow Coma Scale, white blood cell count, hemoglobin, platelet count, prothrombin time-international normalized ratio (PT-INR), serum creatinine, serum total bilirubin, and time from onset to baseline CT scan.

### Image acquisition and segmentation

**Figure 2:**
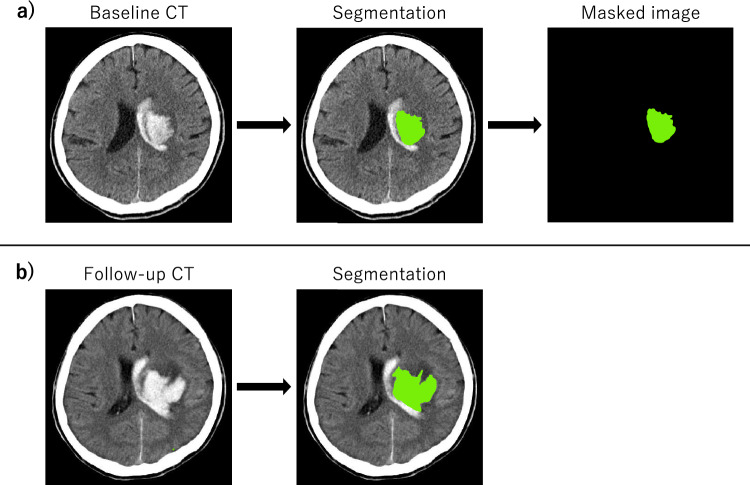
Baseline (**a**) and follow-up (**b**) CT images of a case with hematoma expansion. Intraparenchymal hematomas were manually segmented (green areas), with hematoma volumes computed by planimetry. (**a**) For baseline images, segmented areas were marked as 1 and other areas were marked as 0, which were exported as masked images.

CT scans were performed in the supine position at 120 kVp with a thickness of 0.5–2.0 mm and an image shape of 512 × 512 or greater; the images were exported as original images in the Digital Imaging and Communications in Medicine (DICOM) format. For baseline and follow-up CT scans, intraparenchymal hematomas were manually segmented by two raters, board-certified stroke specialists with more than 15 years of experience, using 3D Slicer, with hematoma volumes calculated by planimetry (Fig. 2). Intraventricular hematomas were neither segmented nor included in the hematoma volume. Hematoma expansion was defined as a volume increase between baseline and follow-up CT scans greater than 6 cm^3^ or 33% of baseline volume^11–13,15,16^. According to this definition, all the patients were labeled as having hematoma expansion or no hematoma expansion. On the baseline CT, segmented areas were marked with a value of 1 and other areas were marked with 0; the images were exported as masked images in DICOM format for the CNN analysis (Fig. 2).

### Evaluation of CT findings at baseline

As CT findings in predicting hematoma expansion, blend sign, intrahematoma hypodensities, and irregular shape were evaluated. Blend sign was defined as the blending of a relatively hypoattenuating area with an adjacent hypoattenuating area within the hematoma^6,7,9^. Intrahematoma hypodensities were defined as the presence of any hypodense region encapsulated within the hematoma and separated from the surrounding parenchyma^7,10^. Irregular hematoma shape was defined as having 2 or more edge irregularities^4,5,7^. These were assessed independently by two raters; prior to assessment, the raters were trained using at least 10 patients with ICH that were not included in this study. In case of disagreement, the findings were reassessed by both raters together until a consensus was reached. Hematoma location and intraventricular hematoma extension were also evaluated.

### Statistical analysis

Categorical variables were summarized as counts with percentages and compared using Fisher's exact test. Continuous variables were summarized as mean with standard deviation or median with interquartile range and compared using Student's t-test or Mann–Whitney U-test, depending on the distribution of the variable assessed by the Shapiro–Wilk test. Sensitivity, specificity, accuracy, and area under the receiver operating curve (AUC) were calculated along with the 95% confidence intervals derived from the actual and predicted labels. P values less than 0.05 were considered significant. Statistical analyses were performed using EZR (Saitama Medical Center, Jichi Medical University, Saitama, Japan)^26^.

### Designing models for prediction

Three types of models were designed to predict hematoma expansion: (1) a CNN model, (2) a multimodal CNN model, and (3) a non-CNN model. A CNN model used CT images as input. A multimodal CNN model used CT images and clinical variables as input, combining CNN analysis of CT images and neural network analysis of clinical variables. A non-CNN model used human-assessed CT findings and clinical variables as input, which were analyzed with machine learning. For each type, several models were devised with different algorithm or input.

**Figure 3:**
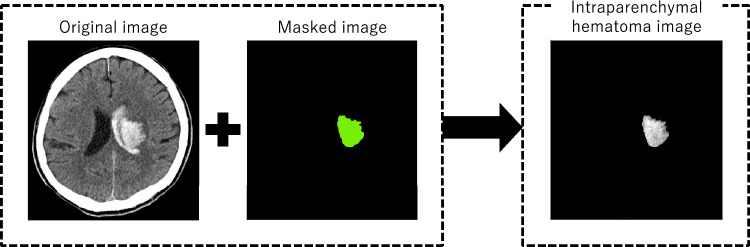
Intraparenchymal hematoma images generated from original and masked images.

CNN Model 1 used unmodified original CT images as input. CNN Model 2 used only intraparenchymal hematoma images, which were generated from original and masked images (Fig. 3).

Multimodal CNN Model 1 used all available clinical variables as one input. For Multimodal CNN Model 2, we first analyzed all clinical variables with univariate analyses between expansion and no expansion cases in the training and validation dataset, and used the clinical variables associated with hematoma expansion with statistical significance. For both models, the original images or the intraparenchymal hematoma images, whichever performed better in the comparison between CNN Model 1 and 2, were used as the other input.

Non-CNN Model 1 and 2 employed k-nearest neighbors as algorithm, while Non-CNN Model 3 and 4 employed fully connected neural network. Non-CNN Model 1 and 3 used all available CT findings and clinical variables as input, whereas non-CNN Model 2 and 4 used CT findings and clinical variables significantly associated with hematoma expansion in univariate analyses between expansion and no expansion cases in the training and validation dataset.

### Preprocessing

All processing was done with Keras (version 2.12.0), a deep learning application programming interface in Python, running with 40 GB of GPU memory. All of the code in this study is available on GitHub (https://github.com/AI-neurosurg/Multimodal-network-for-predicting-hematoma-expansion-in-ICH).

For clinical variables, standardization was first performed for continuous variables in the training and validation dataset. Standardization of the test dataset was then performed based on the mean and standard deviation in the training and validation dataset.

Prior to image processing, all DICOM files were converted to Neuroimaging Informatics Technology Initiative (NIfTI) files. Preprocessing for CNN was performed separately for original and masked images. For the original images, the following steps were executed: (1) density scaling, (2) reslicing, (3) pixel size unification, and (4) resizing. First, after extracting the brain and hematoma by thresholding the Hounsfield units between 0 and 100, the pixel values were scaled between 0 and 1 by dividing the values by 100. Second, the images were resliced with a new slice thickness of 2 mm, and the new number of slices was set to 80, with all slices at or beyond the 81st position from the most cranial slice being deleted. Third, the axial pixel sizes were unified to 0.5 × 0.5 mm, because the image magnification varied between CT scans. Since all image shapes became slightly smaller than 512 × 512, padding was performed to keep the image shape at 512 × 512. Fourth, resizing was done by changing the image shape from 512 × 512 to 256 × 256 to fit the GPU memory. The preprocessed original images were used as input to CNN Model 1. For the masked images, the above steps were executed from the second to the fourth, since the masked images were already binary, either 0 or 1. Intraparenchymal hematoma images were generated from preprocessed original and masked images for CNN Model 2 (Fig. 3).

From the training and validation dataset, 70% were randomly assigned to the training set and the rest to the validation set. To balance the ratio of expansion cases to no expansion cases, data augmentation and random oversampling were applied only to expansion cases in the training set. Data augmentation was conducted in CNN and multimodal CNN models, where images were flipped and rotated 30 degrees. Random oversampling was performed in non-CNN models.

### Model architecture

**Figure 4:**
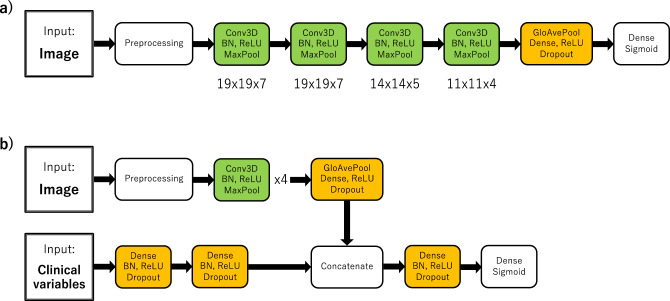
(**a**) The CNN models were composed of four 3-dimensional convolutional layer blocks and one dense layer block, followed by a final dense layer block with sigmoid activation function. The kernel sizes of the convolutional layers were 19 × 19 × 7, 19 × 19 × 7, 14 × 14 × 5, and 11 × 11 × 4, respectively. (**b**) The multimodal CNN models consisted of an image part and a clinical-variables part. The architecture of the image part was the same as in the CNN models (**a**), except for the last block. The clinical-variables part was consisted of two dense layer blocks. These two parts were concatenated, followed by a dense layer block and a final dense layer block.

CNN Model 1 and 2 were composed of four 3-dimensional convolutional layer blocks with batch normalization, ReLU activation function, and max pooling, followed by a dense layer block with global average pooling, ReLU activation function, and dropout (Fig. 4a). At the end, a final dense layer with sigmoid activation function was placed. The kernel sizes in the convolutional layers were 19 × 19 × 7, 19 × 19 × 7, 14 × 14 × 5, and 11 × 11 × 4 consecutively. Multimodal CNN Model 1 and 2 consisted of two parts: an image part and a clinical-variables part (Fig. 4b). The image part had the same architecture as the CNN Models except for the final block. The clinical-variables part was composed of two dense layer blocks with batch normalization, ReLU activation function, and dropout. The image and clinical-variables parts were concatenated in the middle, followed by a dense layer block with batch normalization, ReLU activation function and dropout and a final dense layer with sigmoid activation function.

In non-CNN Model 1 and 2, k-nearest neighbors from scikit-learn machine learning library (version 1.2.2) was adopted. A hyperparameter of the number of neighbors were chosen from 3, 5, 7, 9, and 11. Non-CNN Model 3 and 4 were composed of three dense layer blocks with batch normalization, ReLU activation function, and dropout, followed by a final dense layer block with sigmoid activation function.

The labels to predict in the models were hematoma expansion or no hematoma expansion. No missing data were treated in the models because all the required data, including images and clinical variables, were complete for all patients.

### Training, validation and test

For CNN Models, Multimodal CNN Models, and Non-CNN Model 3 and 4, 70 epochs of training were performed with a batch size of 2, where binary cross-entropy and Adam were used for the loss function and optimizer, respectively^27^. For Adam, the following settings were used: learning rate = 0.001, beta 1 = 0.9, beta 2 = 0.999, and epsilon = 1e-07. The cut-off value of 0.5 was used to binarize the cases. At each epoch, sensitivity and area under the receiver operating curve (AUC) were calculated with the validation dataset to monitor the training process. Five trained model weights from all epochs that had better sensitivity and AUC in validation were selected and used for testing, where the final test result was derived from the weights with the highest sensitivity.

For Non-CNN Model 1 and 2, the training and validation dataset were fitted to the k-nearest neighbors algorithm while changing the hyperparameter of the number of neighbors, to which the test dataset was applied and the final result was derived from the number of neighbors with the highest sensitivity.

### Ethical approval

This study was approved by the following institutional review boards: Mie Chuo Medical Center institutional review board [permit number: MCERB-202321], Matsusaka Chuo General Hospital institutional review board [permit number: 325], Suzuka Kaisei Hospital institutional review board [permit number: 2020-05], and Mie University Hospital institutional review board [permit number: T2023-7]. Because this was a retrospective study, separate informed patient consent was waived by the following institutional review boards: Mie Chuo Medical Center institutional review board [permit number: MCERB-202321], Matsusaka Chuo General Hospital institutional review board [permit number: 325], Suzuka Kaisei Hospital institutional review board [permit number: 2020-05], and Mie University Hospital institutional review board [permit number: T2023-7]. All study protocols and procedures were conducted in accordance with the Declaration of Helsinki. This manuscript was prepared according to the standards for reporting of diagnostic accuracy (STARD) statement.

## Results

After applying the inclusion and exclusion criteria, 273 patients were assigned to the training and validation dataset, while 106 patients were assigned to the test dataset. Patient characteristics of the study population are shown in Table 1. Their row data are stored in OSF in comma-separated values format (https://osf.io/jmnzs).

**Table 1:** Characteristics of the study population.

	Training and validation (n = 273)	Test (n = 106)	P value
Age (years)	71 (59–79)	72 (62–82)	0.099
Sex (male)	159 (57.9)	63 (59.4)	0.817
Medical history			
Hypertension	162 (59.3)	74 (69.8)	0.060
Diabetes mellitus	58 (21.3)	30 (28.3)	0.175
Dyslipidemia	93 (34.1)	37 (34.9)	0.904
Intracerebral hemorrhage	18 (6.6)	7 (6.6)	1.000
Cerebral infarction	30 (11.0)	14 (13.2)	0.593
Ischemic heart disease	15 (5.5)	7 (6.6)	0.633
Anticoagulant use	30 (11.0)	10 (9.4)	0.714
Antiplatelet use	34 (12.5)	25 (23.6)	0.011
Systolic blood pressure (mmHg)	187.6 ± 33.3	182.6 ± 33.6	0.193
Diastolic blood pressure (mmHg)	105.7 ± 23.3	104.2 ± 23.1	0.580
Glasgow Coma Scale	14 (11–15)	15 (12–15)	0.046
White blood cell count (10^6^/mL)	7.60 (5.80–9.60)	7.85 (6.15–9.78)	0.163
Hemoglobin (mg/dL)	13.9 ± 2.1	13.3 ± 1.8	0.005
Platelet count (10^6^/mL)	216.2 ± 59.3	225.1 ± 68.0	0.212
PT-INR	0.96 (0.92–1.02)	0.99 (0.95–1.05)	0.003
Serum creatinine (mg/dL)	0.74 (0.59–0.91)	0.72 (0.58–0.90)	0.589
Serum total bilirubin (mg/dL)	0.7 (0.5–0.9)	0.7 (0.5–1.0)	0.770
Time from onset to baseline CT scan (h)	1 (1–3)	2 (1–4)	< 0.001
CT findings at baseline			
Blend sign	25 (9.2)	5 (4.7)	0.203
Intrahematoma hypodensities	118 (43.2)	33 (31.3)	0.035
Irregular hematoma shape	160 (58.6)	56 (52.8)	0.355
Hemorrhage location			
Brain stem	17 (6.2)	3 (2.8)	0.304
Cerebellum	11 (4.0)	12 (11.3)	0.014
Thalamus	81 (29.7)	35 (33.0)	0.537
Basal ganglia	117 (42.9)	36 (34.0)	0.130
Lobe	47 (17.2)	20 (18.9)	0.764
Intraventricular hematoma extension	103 (37.7)	46 (43.4)	0.349
Baseline hematoma volume, mL	12.2 (5.6–30.2)	10.2 (3.6–19.9)	0.031
Hematoma expansion	54 (19.8)	14 (13.2)	0.179

Data are presented as n (%), mean ± standard deviation, or median (interquartile range).

PT-INR = prothrombin time-international normalized ratio.

On CT findings, intrahematoma hypodensities, hematoma location, and hematoma volume were statistically significant in univariate analyses between expansion and no expansion cases in the training and validation dataset; these were used as input in Non-CNN Model 2 and 4. On clinical variables, anticoagulant use, systolic and diastolic blood pressure, PT-INR, and time from onset to baseline CT were significant and used as input in Multimodal CNN Model 2 and Non-CNN Model 2 and 4.

**Figure 5:**
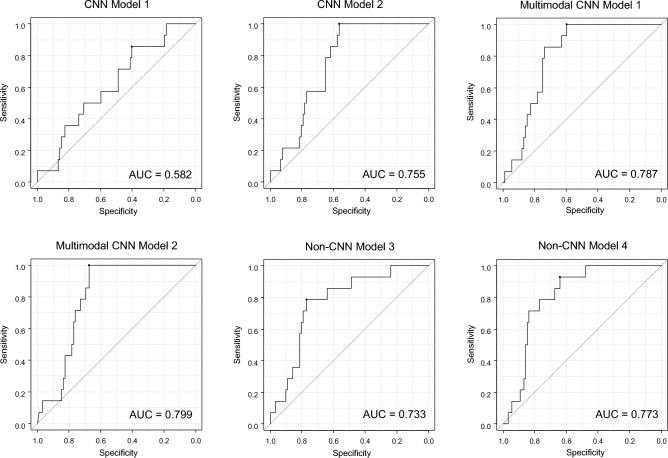
Receiver operating curves for each model in Table 2, except for Non-CNN Model 1 and 2. These models were excluded because they do not return continuous values as a prediction.

The performance of each model is shown in Table 2 and Fig. 5. In CNN Model 1 and 2, sensitivity was the same, but specificity and AUC were higher in CNN Model 2. Therefore, intraparenchymal hematoma images, rather than original images, were used as input of Multimodal CNN Model 1 and 2 (Fig. 3). The number of neighbors of 7 achieved the highest sensitivity for Non-CNN Model 1 and 2.

**Table 2:** Test results for predicting hematoma expansion in each model.

	Sensitivity	Specificity	Accuracy	AUC
CNN Model 1	1.000 (0.768–1.000)	0.163 (0.094–0.255)	0.274 (0.191–369)	0.582 (0.544–0.614)
CNN Model 2	1.000 (0.768–1.000)	0.511 (0.404–0.617)	0.575 (0.476–0.671)	0.755 (0.704–0.807)
Multimodal CNN Model 1	0.857 (0.572–0.982)	0.717 (0.614–0.806)	0.736 (0.641–0.817)	0.787 (0.682–0.893)
Multimodal CNN Model 2	1.000 (0.768–1.000)	0.598 (0.490–0.699)	0.615 (0.552–0.741)	0.799 (0.749–0.849)
Non-CNN Model 1	0.571 (0.289–0.823)	0.761 (0.661–0.844)	0.736 (0.641–0.817)	0.666 (0.525–0.808)
Non-CNN Model 2	0.643 (0.351–0.872)	0.696 (0.591–0.787)	0.689 (0.591–0.775)	0.669 (0.531–0.808)
Non-CNN Model 3	0.857 (0.572–0.982)	0.609 (0.501–0.709)	0.642 (0.543–0.732)	0.733 (0.625–0.840)
Non-CNN Model 4	0.786 (0.493–0.953)	0.761 (0.661–0.844)	0.764 (0.672–0.841)	0.773 (0.653–0.893)

Data are presented as value (95% confidence interval).

AUC = area under the receiver operating characteristic curve.

Sensitivity was higher for CNN Models and Multimodal CNN Models than for Non-CNN Models. In particular, CNN Model 1 and 2 and Multimodal CNN Model 2 achieved a sensitivity of 1.000 (95% confidence interval 0.768–1.000). AUC was above 0.75 for CNN Model 2, Multimodal CNN Model 1 and 2, and Non-CNN Model 2, with the Multimodal CNN Model 2 having the highest AUC. Specificity was highest for Non-CNN Model 1 and 4. Accuracy was highest for Non-CNN Model 4.

Multimodal CNN Model 2 showed the highest sensitivity and AUC of all models. Its model weights are stored in OSF in HDF5 format (419 MB, https://osf.io/wm768).

## Discussion

A multimodal neural network model incorporating CNN analysis of CT images and neural network analysis of clinical variables showed a sensitivity of 1.000 for predicting hematoma expansion in spontaneous ICH. The model outperformed CNN analysis of CT images alone and machine learning analysis of CT findings and clinical variables. It is a highly complete model that utilizes all available patient information. The multimodal model would be beneficial in clinical practice, as it effectively identifies patients who require thorough and intensive care after admission.

For clinicians treating ICH, the greatest concern in predicting hematoma expansion is missing expansion cases because they may experience neurological deterioration and require careful observation in the intensive care units^2,3^. Not missing a single case at risk is critical in stroke care. Therefore, the goal of the prediction in this study was set to achieve higher sensitivity while balancing with AUC. In binary classification, the binary cross-entropy is usually used as the loss function, and training is aimed at minimizing the loss function. However, a low value of binary cross-entropy loss does not always equate to high sensitivity. Thus, for testing, we did not simply select the model weights with the lowest loss value in validation, but selected those with better sensitivity and AUC. However, this selection might have enhanced the model performance.

The Multimodal CNN Model 2, which used CT images and selected clinical variables as input, showed the best performance. The superior performance of Multimodal CNN Model 2 compared to CNN Model 2 underscores the importance of clinical information in predicting hematoma expansion. Here, not all clinical variables were necessary, only 5 were used. The fact that the model works with fewer inputs is critical to its practical use, as it can sometimes be difficult to collect sufficient information in clinical settings. Furthermore, the superiority of multimodal neural network models over non-CNN models underscores that CNN analysis outperforms human-based CT findings evaluation, even when combined with clinical information, in predicting hematoma expansion.

One of the most challenging aspects in the development of CNN and multimodal CNN models was the size of the kernels in the convolutional layers. Typically, a kernel size of 3 or at most 5 is used because a larger kernel size consumes more computational power^28^. However, in preliminary experiments, we observed a divergence in the training process with a kernel size of 3 or 5, even when adding layers or increasing the number of kernels. The voxel size of the CT images was 0.5 × 0.5 × 2.0 mm, where the kernel size of 3 or 5 may have been too small to extract features from the hematoma. The larger kernel sizes up to 19 × 19 × 7 worked effectively in this study; we could not confirm the kernel size in other studies that used CNN to predict hematoma expansion because the programming codes were not disclosed^18–21,29^.

Several considerations have been suggested for the soundness of research using artificial intelligence (AI) techniques, such as the use of an external test set for the final report, transparency of algorithms, etc.^30,31^. However, many clinical studies have not actually followed these basic considerations. In this study, the model was trained on data from several hospitals and tested on external data. Multiple models were created for comparison. Clinical information, algorithm programming code, and model weights were disclosed to make our results verifiable and the model reproducible.

To date, there are 2 studies that predicted hematoma expansion in ICH by analyzing both CT images and clinical information with CNN^29,32^. In one study, hematoma features extracted from CNN and radiomics and clinical variables were integrally analyzed with support vector machine^29^. It achieved sensitivity of 0.83 and AUC of 0.95; however, the testing method was not described in detail and patient data from a single hospital were used for both training and testing^29^. The other analyzed CNN-derived hematoma features and clinical variables using multivariate logistic regression, achieving sensitivity of 0.76 and AUC of 0.83^32^. This is also a single-center study, and the sensitivity is low to use the model in clinical practice. In our study, we achieved a sensitivity of 1.00, which is critical for clinical use in stroke management.

CNN Model 2 using intraparenchymal hematoma images outperformed CNN Model 1 using unmodified original CT images. Therefore, intraparenchymal hematoma images were used as input for multimodal models. However, their segmentations were performed manually by humans in this study because automated segmentation remains unsatisfactory in some cases with an inaccurate differentiation between intraparenchymal and intraventricular hematoma^33–35^. When more accurate segmentation of intraparenchymal hematoma becomes possible, and clinical variables can be automatically collected from the medical record, this prediction task could be fully automated.

Several limitations should be noted. First, although a perfect sensitivity of 1.000 was achieved in a multimodal neural network model while balancing AUC, the lower limit of the confidence interval was 0.768, indicating that more cases are needed for more reliable model validation. Second, although the external dataset from another hospital was used for testing, validation with various patient demographics is required to further ensure the reliability of the developed models. Third, the comparisons of the model performance were not supported by statistical significance testing; instead, simple comparisons were conducted among the models. More cases are also required to statistically demonstrate significant differences based on 95% confidence intervals. Fourth, the images were resized to 256 × 256 to fit the GPU memory. Analysis at the original 512 × 512 size may yield better results. Fifth, only k-nearest neighbors and fully connected neural networks were employed for non-CNN models. Other machine learning models may have performed better, but logistic regression, support vector machines, random forests, and gradient boosting were inferior to k-nearest neighbors in the previous report predicting hematoma expansion^14^. Sixth, this is a retrospective study. Validation with prospective data is required as a future step. Seventh, to apply the models to clinical practice, systems that comprehensively capture patient information, including images and clinical variables, are required. Last, although the clinical variables that are included in the study were generally collected from the patients in the clinical setting for stroke care, they are sometimes unavailable. A model capable of handling missing data may be beneficial.

## Conclusion

We developed a multimodal neural network model incorporating CNN analysis of CT images and neural network analysis of clinical variables to predict hematoma expansion in acute spontaneous ICH. The model was externally validated and outperformed CNN analysis of CT images alone and machine learning analysis of CT findings and clinical variables. The multimodal model achieved sufficient performance with a sensitivity of 1.000 to potentially enable decision support in clinical settings; it effectively identifies patients who require thorough and intensive care after admission. The algorithm programming code and model weights are available for verification and public use. To ensure the reliability of the models, validation with prospective datasets for various patient demographics is necessary as a future step. Furthermore, a model capable of handling missing data or systems that comprehensively capture patient information would be required to enable widespread use of predictive models in clinical practice.

## Data Availability

Row data of patient characteristics are stored in OSF in comma-separated values format (https://osf.io/jmnzs). All code in this study is available on GitHub (https://github.com/AI-neurosurg/Multimodal-network-for-predicting-hematoma-expansion-in-ICH). Model weights of the best-performing multimodal convolutional neural network model are stored in OSF in HDF5 format (419 MB, https://osf.io/wm768).
